# 4q27 deletion and 7q36.1 microduplication in a patient with multiple malformations and hearing loss: a case report

**DOI:** 10.1186/s12920-020-0697-y

**Published:** 2020-03-03

**Authors:** Maolan Wu, Xiangrong Zheng, Xia Wang, Guoyuan Zhang, Jian Kuang

**Affiliations:** Department of Pediatrics, XiangYa Hospital, Central South University, Changsha, China

**Keywords:** 4q deletion, 7q duplication, Developmental delay, Malformation, Pulmonary dysplasia, Hearing disorder

## Abstract

**Background:**

Chromosome deletions of the long arm of chromosome 4 in 4q syndrome are characterized by mild facial and digital dysmorphism, developmental delay, growth retardation, and skeletal and cardiac anomalies, which is regarded as an autism spectrum disorder. Moreover, some scarce reports indicate that patients with 4q interstitial deletion and 7p duplication may present symptoms associated with hearing loss.

**Case presentation:**

A boy with a severe developmental delay not only post-natal but also intrauterine and several dysmorphic features including microcephaly, ocular hypertelorism, exophthalmos, low-set ears, single palmar flexion crease, and overlapping toes presented discontinued cyanosis and recurrent respiratory infections. MRI, BAEP, echocardiogram and bronchoscopy revealed that he had persistent falcine sinus with a thin corpus callosum, left auditory pathway disorder, patent foramen ovale (2 mm), and tracheobronchomalacia with the right superior bronchus arising from the lateral posterior wall of the right main bronchus. Finally, the patient died with severe pneumonia at 10 months. Array CGH revealed a 23.62 Mb deletion at chromosome 4q27, arr [hg19] 4q27-q31.21 (121, 148, 089–144, 769, 263) × 1, and a 0.85 Mb duplication at chromosome 7q36.1, arr [hg19] 7q36.1-q36.2 (152, 510, 685–153, 363,5 98) × 3. It is rare for 4q syndrome cases or 7q duplications previously reported to have a hearing disorder, pulmonary dysplasia, and pulmonary arterial hypertension.

**Conclusions:**

The phenotype of our patient mainly reflects the effects of haploinsufficiency of FGF2, SPATA5, NAA15, SMAD1, HHIP genes combined with a microduplication of 7q36.1.

## Background

Chromosome deletion/duplication is associated with mental disorders and dysmorphism. 4q-syndrome is characterized by chromosomal deletion at the breakpoint 4q31 by Townes and colleagues firstly [1], and was subsequently extended to interstitial and terminal deletions of chromosome 4 [2–5] with an estimated incidence of roughly 1 in 10,000 live births [3, 4]. In the case of chromosomal duplications, the variable phenotypic expressions may vary due to different gene content as a result of the unbalanced rearrangement and the involvement of extrachromosomal material from other chromosomes [6]. The incidence of duplication in the long arm of chromosome 7 is much lower than 4q deletion, and most of the 7q duplicated cases showed unbalanced aberrations resulted from the inheritance of parental balanced chromosomal rearrangements [7–11].

Till now, there is no clinical report on patients with genetic abnormality associated with chromosome 4q deletion and 7q duplication. In this paper, we describe a patient with genetic abnormalities characterized with 23.62 Mb deletion on the long arm of chromosome 4 and a microduplication on the long arm of chromosome 7 presented with hearing impairment, severe developmental delay, and multisystem malformation.

## Case presentation

The child was born as the third child and the first boy to non-consanguineous healthy parents, at completion of 36th weeks of gestation, with a reduced birth weight of 1350 g, length of 35 cm, and head circumference of 28 cm (less than -2SD), borne via cesarean delivery due to the reduction of amniotic fluid. The mother was 30 years old and the father was 36 years old at the time of delivery. Ultrasounds done during pregnancy was normal before the 30th week but indicated significant intrauterine growth retardation afterward. Dexamethasone was prenatally administered to promote lung maturation before delivery. The child suffered from irregular hypopnea immediately after birth and was relieved by positive pressure ventilation within 30 s. After 10 minutes, the child presented obvious respiratory distress syndrome with tachypnea, chest-wall retraction, and cyanosis, and was admitted to NICU for 41 days. However, on successful therapies such as nasal intermittent positive pressure ventilation, traumatic mechanical ventilation, and surfactant replacement therapy dyspnea was relieved. Ultrasonic cardiogram was performed during the first week revealed the patent ductus arteriosus and pulmonary arterial hypertension, for which the sildenafil therapy was initiated. Still, the cyanosis symptoms persisted when the child cried or screamed, and had shown complete hearing loss in the screening tests without congenital cytomegalovirus or syphilis infections. Chromosome analysis revealed 46 XY, 4q deletion.

**Fig. 1:**
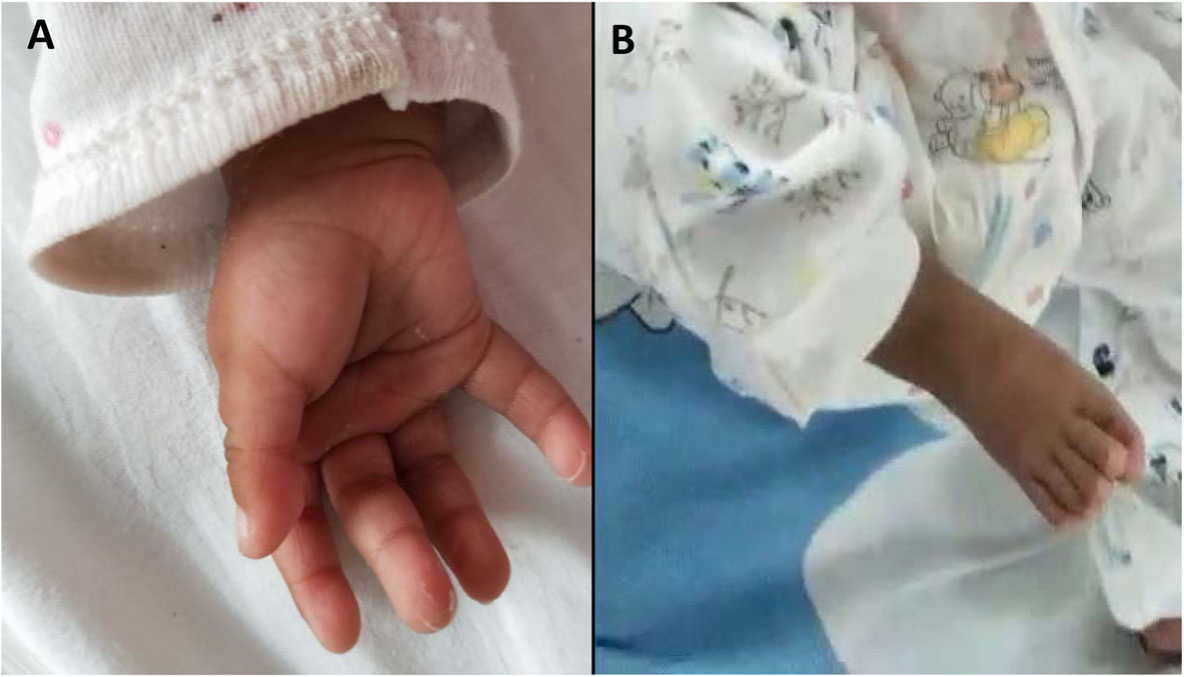
Malformation of hands and feet. A single transverse palmar crease (**a**); The first toe overlapped with the second toe (**b**)

**Fig. 2:**
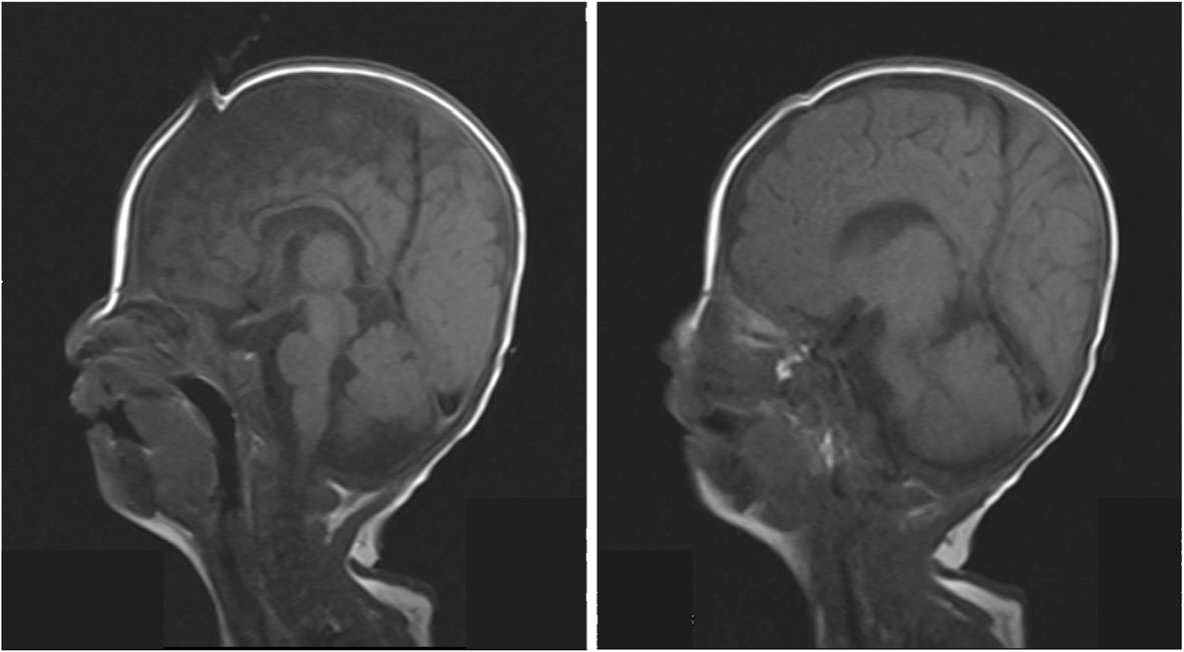
MRI image of head. Persistent falcine sinus and a thin corpus callosum confirmed by MRI

At the age of 3 months, the child was again hospitalized due to aspiration pneumonia and showed exophthalmos, single transverse palmar creases, overlapping toes, left inguinal hernia and severe dystrophy (Fig. 1), and also developed gastroesophageal reflux disease (GERD). MRI showed persistent falcine sinus with a thin corpus callosum (Fig. 2). Brainstem auditory evoked potential (BAEP) was tested at 4 months of age which revealed the left auditory pathway disorder showing no reaction to clicking sounds ranging from 30 to 120 dB. However, the right auditory pathway reaction was well with a BAEP threshold of 30 dB. Bronchoscopy showed tracheobronchomalacia (TBM) and the right superior bronchus arising from the lateral posterior wall of the right main bronchus. Ultrasonic cardiogram showed patent foramen ovale (2 mm) and normal pressure in the pulmonary artery. CT and CTA of the heart were performed which showed normal results. Metabolic diseases screened with serum amino acids and urine organic acids excluded congenital disorders, but all yielded normal results. During hospitalization, the child gained weight nearly 1 kg per month when fed on Alfaré, but was stopped due to family financial crisis and the weight growth speed reduced drastically to a low level of 5.3 kg at 8 months (less than -3SD). Finally, on reaching 10 months of age the child could not recover from cyanosis and died due to severe pneumonia.

### Chromosomal microarray analysis

**Fig. 3:**
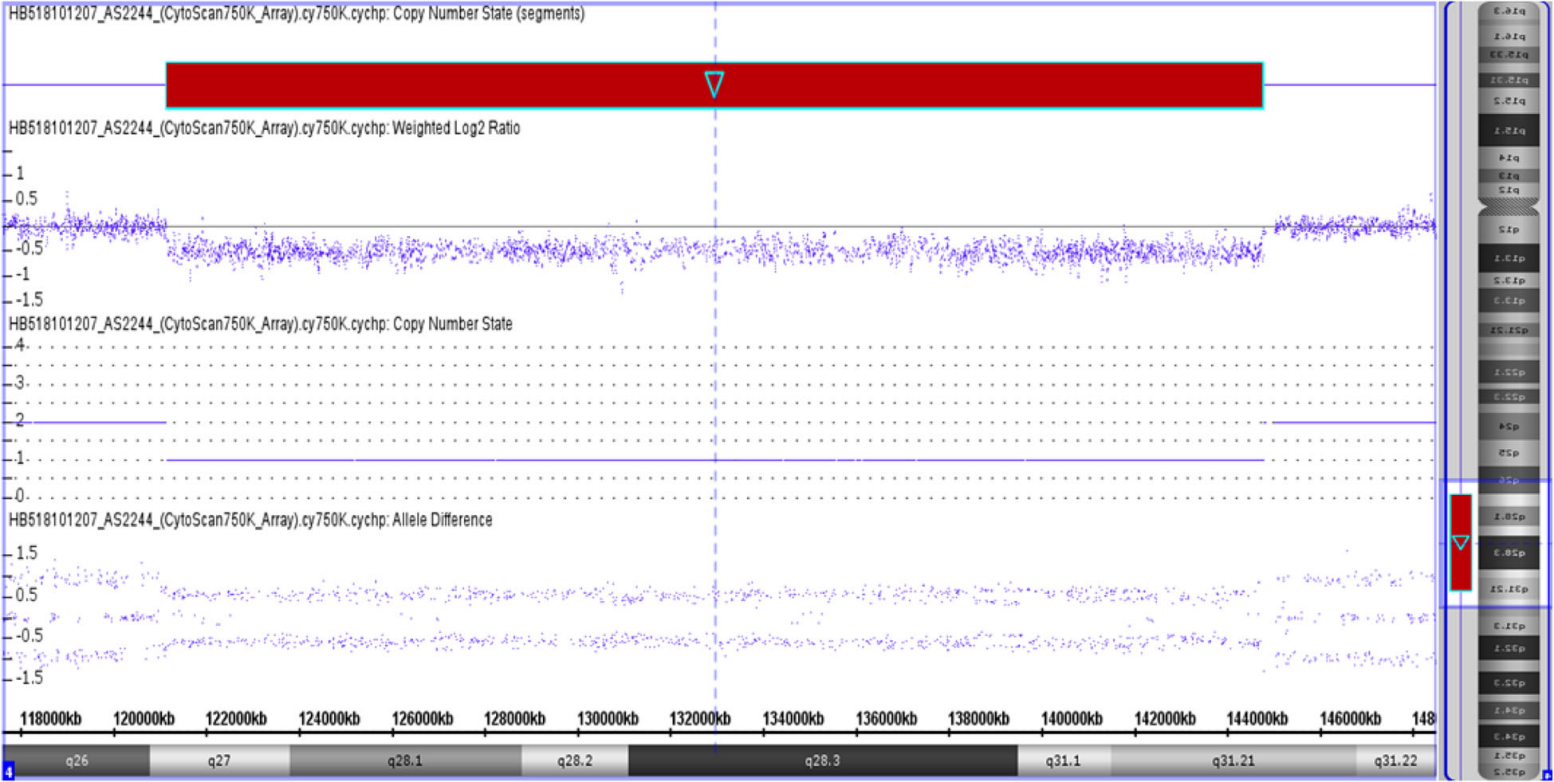
Red is the deletion at the long arm of chromosome 4

**Fig. 4:**
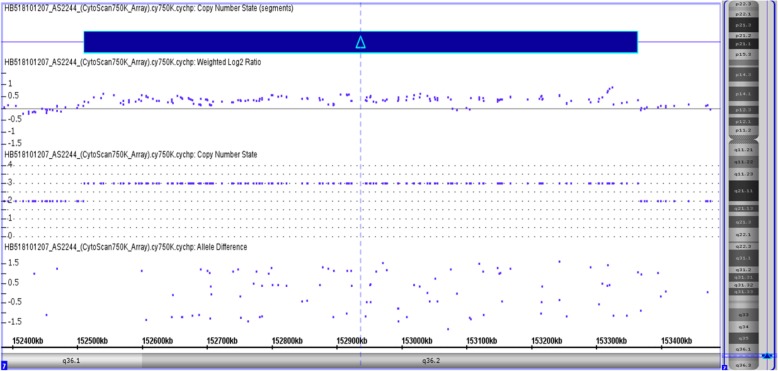
Blue is the duplication at the long arm of chromosome 7

CMA (Chromosomal microarray analysis) was performed using SurePrint G3 customized array (Agilent Technologies, Santa Clara, CA, USA). Previously validated platform settings were consistently utilized for CNV detection and filtering. CNVs within the size range of 2–400 kb were detected via CMA and were further confirmed by manual inspection. It was revealed that there were 23.62 Mb deletion and 0.85 Mb microduplication at chromosome 4q27, arr[hg19] 4q27-q31.21 (121, 148, 089–144, 769, 263) × 1 (Fig. 3), and chromosome 7q36.1, arr[hg19] 7q36.1-q36.2 (152, 510, 685–153, 363, 598) × 3 (Fig. 4), respectively. Moreover, it was evident that within this deleted region there were 117 genes (64 listed in OMIM), and 10 genes listed in OMIM span over the duplicated region.

### Whole-exome sequencing

Genomic DNA samples were extracted from the patient’s peripheral blood using QIAamp® Blood Mini Kit (Qiagen, Hilden, Germany). DNA target regions were captured by hybridizing the genomic DNA sample library with the Agilent SureSelect Human All Exon V5 (Agilent, USA). The captured and amplified DNA samples were sequenced using Illumina NovaSeq6000 (Illumina, San Diego, CA, USA) with 150 base-paired end reads.

The bioinformatics analysis of the raw data according to ‘Standards and Guidelines for Validating Next-Generation Sequencing Bioinformatics Pipelines’ [12], include the following steps: Sequence Generation, Sequence Alignment, Variant Calling (SNV, INDEL, CNV), Variant Filtering, Variant Annotation, and Variant Prioritization. Indeed, we could find a deletion at chromosome 4q27 (chr:121302077–144,797,407) × 1, but could not get any other significant information from the results and analysis of WES to explain the real cause of hearing disorder of the child.

## Discussion and conclusions

According to a literature search, there were no previous reports describing any case with deletion of the long arm of chromosome 4 and duplication of the long arm of chromosome 7. Even though the coordinates of the deletions have varied, our patient shared many clinical features with patients who had deletions in the similar region [13–15] (Table 1). All of them presented facial dysmorphism and developmental delay, which have reported that 99 and 94% of patients with 4q deletions presented these features respectively [4].

**Table 1:** Comparison of demographic and phenotypic characteristics of our patient with other subjects reported in previous studies

Cytogenetic results	Age	Sex	Parental studies	Growth		Dysmorphism		Central nervous system	Intellectual disability	Cardio-vascular	Gastro-intestinal	Renal /genitourinary	Hearing impairment	Reference
				IUGR	Post-natal growth retardation	Facial	Digital							
4q del:121,148,089-144,769,263; 7q dup:152,510,685-153,363,598	1 y	M	–	+	+	+	+	+	+	+	+	–	The left hearing loss	Our patient.
4q del:122,756,085-128,434,447	12 y	M	+		+	+	–	+	+	+	+	+	–	Hickey et al. [14]
4q del:136,127,048 - 150,690,325	9 m	F			+	+	–	+	+	+	+	+		Duga et al. [15]
4q del:111,310,828–130,503,896	3 d	F	–	–	–	+	+	–		+			–	Strehle et al. [13]
4q del:113,517,078–130,278,522	2 y	M	–	–	+	+	+	+		–			–	
4q del:127,979,585–140,587,349	33 y	F	Mother: inv. (9)p11q13		+	+	+	+		+	+		–	
4q del:184,046,156-190,901,117	8 y	M	*–*	–	+	+		–	–	+	–	+	Bilateral hearing impairment (60 dB)	Vona et al. [16]
deletion 4q33 → q35	11 m	M		–	–	+	+						Mild bilateral conductive deafness	Calabrese et al. [17]
7q del:148,472,027–157,265,994; dup:138,293,371–148,443,994	3 y	F	*–*	+	+	+	+	+	+	–	+	–	Bilateral hearing impairment (60 dB)	Pavone et al. [18]
inv(7) (q22.1 q31.2) t (7;8) (q21.3 q22.1; q23.3 q24.12)	5 y	F	*–*	–	–	+	+	+	+	+	–	–	Severe bilateral hearing impairment	Bernardini et al. [19]

Unilateral hearing loss is more common than bilateral [20], and it was previously reported that more than one out of ten children initially diagnosed with unilateral hearing loss will progress to bilateral hearing loss [21–23]. Cochlear nerve deficiency is the most common type of malformation observed in the setting of congenital unilateral hearing loss [24–26]. Although there are reports of familial unilateral hearing loss [27–30], genetic mutations associated specifically with unilateral hearing loss have yet not been identified with certainty [31]. There are 4 reports of 4q deletions or 7q duplications with hearing impairment available in the literature. The four cases included a 8-year-old boy with deletion in 4q35.1q35.2 region [16], a male infant with deletion in 4q33q35 [17], a 3-year-old girl with duplication of 7q34q35 and deletion in 7q36 [18], and a girl with Complex rearrangement of 7q21.13-q22.1 [19], who were all having bilateral hearing loss with low-set ears (Table 1). Reviewing 141 cases in DECIPHER database showed that only a girl (DECIPHER ID: 293597) with mutations in *SPATA5* (located in 4q28.1) and TSHR presented sensorineural hearing impairment.

SPATA5, also known as a spermatogenesis-associated factor (SPAF), was thought to express subcellular in the spermatogonia and spermatocytes, and was associated with mitochondrial function [32]. But the following studies of *SPATA5* have suggested a role of the *SPATA5* gene not only in neuronal development but also in spermatogenesis. It was dominantly cytosolic in cortical neurons [33–36]. The SPATA5 deficiency affects mitochondrial morphology and inhibits mitochondrial dynamics, delays neuronal development, and is also associated with decreased cellular ATP [36]. All the patients with *SPATA5* variants reported in the literature so far have presented with developmental delay starting in early infancy, 77% presented sensorineural hearing loss, 73% suffered from gastrointestinal problems such as GERD and feeding problem, and 67% was revealed with abnormal brain MRI including hypoplasia of corpus callosum [36].

Furthermore, the deletion of fibroblast growth factor-2 (*FGF2*) might act an important role in our patient’s phenotype. *FGF2* has a haploinsufficiency score (HI index) of 1.68% indicating a highly likely chance to exhibit haploinsufficiency. It plays an important role in the regulation of cell survival, cell division, angiogenesis, cell differentiation, and cell migration and reaches high concentrations in the brain and pituitary. Moreover, it encodes a kind of protein that is a member of the fibroblast growth factor (FGF) family which is not only implicated in limb development, wound healing and tumor growth [37], but also stimulates proliferation of neuronal precursor cells isolated from different regions of the developing central nervous system [38]. FGF signaling is critically required for the in vivo induction of the otic placode during embryonic inner ear development [39]. It is proved that *FGF2* could induce the proliferation and survival of auditory neuroblasts in murine [40].

The *NAA15* gene located at 4q31.1 involved in our patient’s deletion region has been proved to encode a component of the Nat A N-acetyltransferase complex, which is essential for normal cell function in humans, tethering the complex to the ribosome for posttranslational modification of proteins as they exit the ribosome [41]. Cheng et al. [42] proved that haploinsufficiency, patients with copy-number variation (CNV) deletions involving *NAA15* and surrounding genes can lead to mild intellectual disability, mild dysmorphic features, motor delays, growth retardation through identifying and phenotypically characterizing 38 individuals with different likely gene disruption (LGD) variants in *NAA15* that is followed by functional assays in yeast.

In addition to *SPATA5*, *FGF2*, and *NAA15,* the deficiency of *SMAD1* may also play a role in the development of pulmonary hypertension [43–45],. and the *HHIP* possibly might have effected the lung malformation of our patient [46, 47].

In conclusion, we report a boy with a 23.62 Mb of 4q deletion and a 0.85 Mb of 7q duplication, suffered from severe developmental delay, and dysmorphic features similar to other patients of 4q deletion or 7q duplication. But his bronchial deformity, pulmonary arterial hypertension, especially unilateral hearing loss seems to be very unusual. The deletion of the region between 4q27-q31.21 and the duplication between 7q36.1-q36.2 have affected some genes leading to exhibit haploinsufficiency and resulted in these clinical symptoms. The deficiency of *SPATA5 and FGF2* could give a possible explanation for the unilateral hearing loss. In the future, the molecular genetic techniques by combining transcriptomic and proteomic methods with array CGH, it would be possible to precisely examine this region to understand the complex genomic characterization leading to various pathophysiological abnormalities.

## Data Availability

The datasets (whole-exome sequencing, chromosomal microarray, and Sanger sequencing files) used and/or analyzed during the current study are available from the corresponding author on reasonable request.

## Abbreviations

BAEP: Brainstem auditory evoked potential
CGH: Comparative genomic hybridization;
CMA: Chromosomal microarray analysis
CT: Computed tomography
CTA: Computed tomography angiography
GERD: Gastroesophageal reflux disease
IUGR: Intrauterine growth retardation
MRI: Magnetic resonance imaging
